# *In vitro* Chicken Bone Marrow-Derived Dendritic Cells Comprise Subsets at Different States of Maturation

**DOI:** 10.3389/fimmu.2020.00141

**Published:** 2020-02-26

**Authors:** Robin H. G. A. van den Biggelaar, Ger J. A. Arkesteijn, Victor P. M. G. Rutten, Willem van Eden, Christine A. Jansen

**Affiliations:** ^1^Department of Biomolecular Health Sciences, Division of Infectious Diseases and Immunology, Faculty of Veterinary Medicine, Utrecht University, Utrecht, Netherlands; ^2^Department of Veterinary Tropical Diseases, Faculty of Veterinary Science, University of Pretoria, Pretoria, South Africa

**Keywords:** chicken, bone marrow-derived dendritic cells, maturation, GM-CSF, IL-4, MHC-II

## Abstract

Research in chickens has been fundamental for the discovery of basic aspects of the immune system and has led to an interest in the in-depth characterization of avian immune cell types including dendritic cells (DCs). The *in vitro* generation and expansion of chicken bone marrow-derived DCs (chBMDCs) in the presence of granulocyte-macrophage colony-stimulating factor (GM-CSF) has provided a way to study chicken DCs, which are only present at limited cell numbers *in vivo*. This method has been employed to study the interactions between chicken DCs and pathogens or vaccines. However, a detailed characterization of the chBMDC culture is still lacking. In the present study, we performed an elaborate phenotypical and functional analysis of the chBMDC culture and addressed its heterogeneity. After 8 days of culture, chBMDCs comprised major histocompatibility complex class II (MHC-II)^low^ and MHC-II^high^ subsets with different morphologies. Compared with MHC-II^low^ chBMDCs, the MHC-II^high^ subset showed a more mature phenotype, with higher expressions of CD1.1, CD40, CD80, CCR7, and CD83, and a relatively low opsonophagocytic capacity. Nevertheless, MHC-II^high^ chBMDCs did not show an increased capacity to induce T-cell proliferation. Therefore, MHC-II^high^ chBMDCs were found to be semi-mature. Interestingly, the presence of the semi-mature MHC-II^high^ chBMDC subset reduced when cells were cultured in the presence of IL-4. Finally, prolonged cell culture after fluorescence-activated cell sorting (FACS) converted the semi-mature MHC-II^high^ subset back into the immature phenotype of the MHC-II^low^ subset, demonstrating plasticity of their maturation state. This detailed characterization explained the heterogeneity of the chBMDC culture by the simultaneous presence of immature and semi-mature chBMDC subsets, in addition to cells without features of antigen-presenting cells. Our findings are instrumental for the interpretation of experiments using the chBMDC culture in past and future research by providing insights into its phenotypically and functionally distinct cell types.

## Introduction

Dendritic cells (DCs) are important innate immune cells that capture and process antigens to present them to cells of the adaptive immune system (1). Adaptive immune responses result in pathogen-specific and long-lasting immunological memory, which enables the immune system to act more rapidly upon a second encounter with the pathogen. Vaccination against pathogens critically depends on DCs, which, respectively, support and fine-tune antigen presentation by co-stimulatory molecules and cytokines. The expression pattern of co-stimulatory molecules and cytokines by DCs depends on signals from their environment, including pathogen-associated molecular patterns (PAMPs) (1, 2), damage-associated molecular patterns (DAMPs) (3), and cytokines (1, 2, 4).

DCs are rare cells in all tissues and hard to isolate, which makes the use of primary DCs in functional assays challenging. To overcome this problem, granulocyte-macrophage colony-stimulating factor (GM-CSF)-differentiated bone marrow-derived DC (BMDC) culture methods have been developed to generate DCs in large numbers (5). The availability of cultured BMDCs facilitates their use in *in vitro* screening methods, including immunogenicity tests for vaccines and toxicity tests for allergens (6–8). In addition to well-characterized murine BMDC culture methods, such methods have been developed for veterinary species, including dogs [Ricklin (9)], cats (10), cattle (11), sheep (12), pigs (13), and chickens (14).

Chicken BMDCs (chBMDCs) have been cultured in the presence of both recombinant chicken GM-CSF and interleukin-4 (IL-4) and were defined as DCs because of their typical stellate morphology and high expression of both major histocompatibility complex class II (MHC-II) and CD11b/c (14). This chBMDC culture method has led to several studies into the role of chicken DCs in infection and vaccination. Maturation of chBMDCs has been observed after stimulation with lipopolysaccharide (LPS) or CD40L, as demonstrated by increased surface expression of co-stimulatory molecules CD40, CD83, and CD86; reduced phagocytosis and endocytosis; and an increased ability to induce a mixed lymphocyte reaction (14). Similarly, chBMDCs have been found to mature upon exposure to avian influenza virus (15, 16), infectious bursal disease virus (17), or *Salmonella enteritidis* and *Salmonella gallinarum* vaccine candidates (18, 19).

Despite the widespread use of BMDCs originating from chickens and other species, a recent transcriptome study showed that murine GM-CSF-differentiated BMDCs differ phenotypically from murine DC populations *in vivo* (20). Moreover, this study revealed that murine BMDC cultures comprise both CD11b^high^ MHC-II^low^ macrophage-like and CD11b^low^ MHC-II^high^ DC-like subsets that are closely related, but still phenotypically and functionally different. These findings had implications for conclusions drawn using *in vitro* murine BMDC cultures as a model for DC biology *in vivo* and are part of the ongoing discussion on how to distinguish DCs and macrophages (20–25). In addition, these findings stressed the importance of thorough characterization of the cellular subsets present in *in vitro* BMDC cultures and triggered us to explore in depth the nature of chBMDCs raised *in vitro* with GM-CSF and to determine whether these indeed represent DC-like cells.

The initial results of the present study showed that the chBMDC culture was heterogeneous and comprised MHC-II^low^ and MHC-II^high^ subsets, similar to observations in murine BMDC cultures. Therefore, we hypothesized that chBMDC culture comprised MHC-II^low^ macrophage-like and MHC-II^high^ DC-like subsets. However, in contrast to murine BMDC cultures, the MHC-II^low^ and MHC-II^high^ subsets of the chBMDC culture were found to reflect different maturation states rather than distinct cell types. MHC-II^high^ chBMDCs were found to exhibit increased expression of costimulatory molecules, also in the absence of stimuli. These findings on chBMDCs may have important consequences for conclusions drawn in past and future studies that make use of the chBMDC culture as a model for *in vivo* DC biology in chickens, in particular studies that assess chBMDC maturation.

## Materials and Methods

### Bone Marrow Isolation

Eighteen-day-old embryonated NOVOgen Brown eggs were obtained from a commercial breeder (Verbeek Broederij, Zeewolde, the Netherlands). Chicken embryos were removed from the eggs and euthanized by decapitation. Next, the tibiae and femurs were collected, bone heads were removed, and bone marrow was harvested by flushing the bones with RPMI-1640 cell culture medium supplemented with GlutaMAX™-I, phenol red, and HEPES (Gibco™, Life Technologies Limited, Paisley, UK) under sterile conditions using a Plastipak™ 10-ml syringe with a Microlance™ 3 21-G needle (both from BD Biosciences, Pharmingen, San Diego, CA, USA). Bones and bone marrow cells were kept on ice during the whole procedure. Bone marrow cells from 200 embryos were pooled, gently squeezed through a Falcon® 70-μm cell strainer (Corning®, Corning B.V. Life Sciences, Amsterdam, the Netherlands), and stored at −140°C in RPMI, 50% chicken serum (Gibco™, Life Technologies Limited, Paisley, UK), and 10% DMSO (Honeywell, Bucharest, Romania). This procedure resulted in batches comprising 1.3–2.3 × 10^9^ bone marrow cells, which were frozen at a concentration of 2.5–5 × 10^7^ cells per cryotube.

### chBMDC Culture

As previously described by others (26), chBMDCs were cultured from isolated bone marrow cells in RPMI-1640 cell culture medium supplemented with 5% chicken serum and 50 U/ml of penicillin–streptomycin (all from Gibco™, Life Technologies Limited, Paisley, UK) in the presence of recombinant GM-CSF (and IL-4) at 41°C, 5% CO_2_. Recombinant GM-CSF and IL-4 were produced using COS-7 cells transfected with pCI-neo (Promega Corporation, Madison, Wisconsin, USA) expressing the relevant cytokine, which were a kind gift from P. Kaiser and L. Rothwell (Roslin Institute, Edinburgh, UK). The concentrations of the recombinant cytokines are given as a dilution of supernatant from transfected COS-7 cultures in accordance with a previous study (27). GM-CSF was used at the titrated concentration (2 μl/ml) that resulted in the highest percentage of MHC-II^+^ CD40^+^ CD80^+^ cells. In one experiment, the chBMDC culture was supplemented with GM-CSF and titrated concentrations of IL-4. Bone marrow cells were seeded at 2.5 × 10^6^ cells per milliliter in 75-cm^2^ cell culture flasks in 15 ml of RPMI-1640 medium per flask, in 25-cm^2^ cell culture flasks in 5 ml of RPMI-1640 medium per flask, in Costar® six-well plates in 2 ml of RPMI-1640 medium per well, or in Costar® 24-well plates in 0.5 ml of RPMI-1640 medium per well (all from Corning®, Corning B.V. Life Sciences, Amsterdam, the Netherlands) depending on the required sample size. Early in the morning at day 3, culture medium with non-adherent cells was removed, and fresh RPMI-1640 medium with GM-CSF (and IL-4) was added. Late in the afternoon at day 4, the cultures received another volume of RPMI-1640 medium with GM-CSF (and IL-4). The morphology of chBMDCs was examined by light microscopy using an EVOS FL microscope (AMG, Mill Creek, Washington, USA). In selected experiments, chBMDC cultures were matured by 100 ng/ml of LPS O127:B8 (Sigma-Aldrich, Saint Louis, MO, USA) stimulation for 24 h at day 7. To harvest the cultures at day 8, the medium with non-adherent cells was first collected. Subsequently, loosely adherent cells were washed and collected with Dulbecco's phosphate-buffered saline (DPBS) without calcium and magnesium (DPBS^−/−^; Lonza, Basel, Switzerland). Finally, the remaining adherent cells were incubated in DPBS^−/−^ supplemented with 5 mM UltraPure EDTA (Invitrogen™, Life Technologies Europe BV, Bleiswijk, the Netherlands) for 10 min at room temperature (RT) before being collected as well. All cell-containing fluids (cell culture medium, DPBS^−/−^, and DPBS^−/−^ 5 mM EDTA) obtained during the harvest procedure were pooled for subsequent experiments.

### Flow Cytometry Analyses and Antibodies

Antibodies and streptavidin conjugates used in this study are listed in Table 1. All were diluted in fluorescence-activated cell sorting (FACS) buffer, containing DPBS^−/−^ + 0.5% BSA and 0.005% NaN_3_ (both from Sigma-Aldrich, Saint Louis, MO, USA), which was used for staining and washing steps. Antibodies and streptavidin conjugates were used at titrated concentrations to stain 0.5–1.0 × 10^6^ freshly harvested chBMDCs per 50 μl for 20 min at 4°C. Between staining steps, chBMDCs were washed twice with FACS buffer. To assess viability, the cells were first washed in DPBS^−/−^ and then stained with Zombie Aqua Fixable Viability Dye (BioLegend Inc., San Diego, CA, USA) diluted in DPBS^−/−^ for 20 min at 4°C. From each sample, 50,000–100,000 chBMDCs were analyzed using a CytoFLEX LX flow cytometer, equipped with 375-, 405-, 488-, 561-, 638-, and 808-nm lasers (Beckman Coulter Inc., Brea, CA, USA), FlowJo Software v. 10.5 (FlowJo LCC, Ashland, OR, USA), and Prism 7 (GraphPad Software Inc., San Diego, CA, USA).

**Table 1:** Antibodies used in this study.

**Antibody name**	**Isotype**	**Clone**	**Target**	**Figure**	**Source**
MαCh–MHC-II Alexa Fluor 488	IgG1	2G11	MHC-II	1	SouthernBiotech
MαCh-Ia PE	IgM	Cia	MHC-II	2, 6, 7, 9	
MαCh-Ia UNLB	IgM	Cia	MHC-II	3	
MαCh–β_2_-microglobulin UNLB	IgG1	F21-21	MHC-I	2, 6	
MαCh–monocyte/macrophage UNLB	IgG1	KUL01	MRC1L-B	2, 6	
MαCh–c-kit BIOT	IgG2a	Kit2c75	c-kit, CD117	2, 6	
MαCh-CD1.1 UNLB	IgG1	CB3	CD1.1	2, 6	
M IgM, κ isotype Ctrl PE	IgM	MM-30	-	2, 6	BioLegend
MαCh–CD40 UNL	IgG2a	AV79	CD40	2, 6	Bio-Rad
MαCh–CD80 UNLB	IgG2a	IAH:F864DC7	CD80, B7-1	2, 3, 6, 7, 9	
MαCh–CSF1R UNLB	IgG1	ROS-AV170	CSF1R, CD115	2, 3, 6, 7, 9	
MαCh−5C7 UNLB	IgG1	5C7	Putative CD11b/c^*^	2, 6	Produced in house^†^
RαM–IgG1 BV421	IgG	RMG1-1	Mouse IgG1	7, 9	BioLegend
Streptavidin APC	-	-	Biotin	2, 6	BD Pharmingen
GαM-IgG1 APC	IgG	-	Mouse IgG1	2, 6	SouthernBiotech
GαM-IgG2a APC	IgG	-	Mouse IgG2a	2, 6, 7, 9	
GαM-IgM Alexa Fluor 488	IgG	-	Mouse IgM	3	
GαM-IgG2a Alexa Fluor 568	IgG	-	Mouse IgG2a	3	Invitrogen
GαM-IgG1 Alexa Fluor 647	IgG	-	Mouse IgG1	3	

*M, mouse; Ch, chicken; G, goat; R, rat; MHC-II, major histocompatibility complex class II; MRC1L-B, mannose receptor C-type 1-like B; CSF1R, colony-stimulating factor 1 receptor; APC, allophycocyanin; PE, R-phycoerythrin; BV421, Brilliant Violet 421*.

* *It is yet uncertain whether MαCh-5C7 recognizes CD11b or CD11c*.

† *Hybridoma was a gift from T.W. Göbel (Ludwig Maximilians Universität, Munich, Germany). Sources: SouthernBiotech, Birmingham, AL, USA; Bio-Rad Laboratories B.V., Veenendaal, the Netherlands; BioLegend Inc., San Diego, CA, USA; Invitrogen^†^, Life Technologies Europe BV, Bleiswijk, the Netherlands; BD Biosciences, Pharmingen, San Diego, CA, USA*.

### Immunofluorescence Microscopy Analyses

Ethanol-cleaned 12-mm glass coverslips (Waldemar Knittel Glasbearbeitungs GmbH, Brunswick, Germany) were placed into the wells of a 24-well cell culture plate. Next, bone marrow cells were seeded and cultured in complete RPMI culture medium in the presence of recombinant GM-CSF at 41°C, 5% CO_2_. At day 8, differentiated chBMDCs on glass coverslips were either washed three times with DPBS^−/−^ or first stained with fluorescently labeled lectin wheat germ agglutinin (WGA)-Alexa Fluor 488 (Invitrogen™, Life Technologies Europe BV, Bleiswijk, the Netherlands). Staining with WGA-Alexa Fluor 488 was performed by washing the cells twice with cold DPBS with calcium and magnesium, followed by staining with WGA-Alexa Fluor 488 diluted in DPBS with calcium and magnesium for 20 min at 4°C. Fixation was performed in DPBS^−/−^ with 4% paraformaldehyde (Alfa Aesar, Haverhill, MA, USA) for 30 min at RT. Subsequently, the fixatives were quenched by washing the fixed samples three times with DPBS^−/−^ and 10 mM glycine (Merck Millipore, Burlington, MA, USA) and blocked in blocking buffer, containing DPBS^−/−^, 0.05% Tween-20 (Sigma-Aldrich, Saint Louis, MO, USA), and 2% bovine serum albumin (Sigma-Aldrich, Saint Louis, MO, USA), overnight at 4°C. The coverslips were stained with the cells faced-down on Parafilm in 25 μl of blocking buffer with primary antibodies for 2 h and secondary antibodies for 1 h at RT (antibodies are listed in Table 1). In addition, a nuclear staining was performed in 25 μl of blocking buffer at 10 μg/ml with 4′,6-diamidino-2-phenylindole (DAPI) (Sigma-Aldrich, Saint Louis, MO, USA) for 5 min at RT. Between staining steps, the samples were washed three times with DPBS^−/−^ with 0.05% Tween-20. The last wash step was performed in distilled water, before mounting the samples on Polysine® microscope slides (Menzel Glaser GmbH & Co KG, Braunschweig, Germany) in a FluorSave reagent (Calbiochem®, Merck Millipore, Burlington, MA, USA). The samples were captured using a TCS-SPE-II confocal microscope (Leica Microsystems B.V., Amsterdam, the Netherlands) equipped with 405-, 488-, 561-, and 635-nm diode lasers and processed using Fiji software (28).

### Phagocytosis of IgY-Opsonized Beads by chBMDC Subsets

Chicken serum IgY fraction (Agrisera AB, Vännäs, Sweden) was added at 14.4 mg/ml to 1.44 × 10^10^ beads per milliliter of 1-μm crimson carboxylate-modified FluoSpheres (Invitrogen™, Life Technologies Europe BV, Bleiswijk, the Netherlands) and incubated overnight on an orbital shaker at 4°C to create IgY-opsonized beads. The next day, the beads were washed twice and resuspended in DPBS^−/−^ with centrifugation steps at 3,000 *g* for 20 min at 4°C in between. To confirm IgY coupling, the beads were stained in FACS buffer with MαCh IgY-PE (SouthernBiotech, Birmingham, AL, USA) and analyzed on the CytoFLEX LX flow cytometer (*data not shown*). Next, the IgY-coupled beads were used in a phagocytosis assay to assess bead uptake by chBMDCs. After 8 days of culture in a 24-well plate, chBMDCs from one well were harvested and counted to determine the number of IgY-opsonized beads needed to obtain a 1:1 bead-to-cell ratio. Next, crimson beads were added to the remaining wells followed by 4-h incubation at 41°C, 5% CO_2_, to allow phagocytosis by chBMDCs. Subsequently, chBMDCs were harvested and stained for flow cytometry or confocal microscopy according to the methods described above. For flow cytometry, the cells were stained for MHC-II expression and viability, using Zombie Aqua Fixable Viability Dye, and analyzed using the CytoFLEX LX flow cytometer. For confocal microscopy, the cells were stained with WGA-Alexa Fluor 488, MαCh-Ia BIOT, and streptavidin Alexa Fluor 405 and analyzed using the TCS-SPE-II confocal microscope.

### IL-4 Bioactivity Assessment by ^3^H-Thymidine Incorporation by PBMCs

The ^3^H-thymidine incorporation assay to measure IL-4 bioactivity was modified from a published method to assess peripheral blood mononuclear cell (PBMC) proliferation (27). Heparinized blood was collected from healthy chickens (under registration number AVD108002016642-1 from the Dutch Central Authority for Scientific Procedures on Animals). The chickens were daily monitored by animal caretakers for signs and symptoms of disease, which were absent for the chickens used in this study. PBMCs were isolated from heparinized blood by density gradient separation using Ficoll-Paque PLUS (GE Healthcare, Chicago, IL, USA) according to standard procedure. Collected PBMCs were resuspended in 2 ml of Iscove's modified Dulbecco's medium (IMDM) culture medium supplemented with GlutaMAX™-I, phenol red, and HEPES, with 8% fetal bovine serum (FBS), 2% chicken serum, and 50 U/ml of penicillin–streptomycin (all from Gibco™, Life Technologies Limited, Paisley, UK). The cells were counted and seeded in a 96-well flat-bottom culture plate with 100 μl of IMDM medium per well containing 2 × 10^5^ cells. The cells received different concentrations (ranging 1:25–1:250) of cell culture supernatant from COS-7 cells transfected with an IL-4 or empty pCI vector and were incubated for 4 days at 41°C, 5% CO_2_. Subsequently, 0.4 μCi of ^3^H-thymidine per well was added to the culture for 18 h, and the cells were harvested using a Harvester 96 (TOMTEC Imaging Systems GmbH, Unterschleißheim, Germany). ^3^H-Thymidine incorporation by the cells was determined in a 1,450 MicroBeta Plus liquid scintillation counter (Wallac, PerkinElmer Life Sciences, Zaventem, Belgium).

### Separation of chBMDC Subsets by FACS

For sorting, chBMDCs were stained with antibodies specific for MHC-II, colony-stimulating factor 1 receptor (CSF1R), and CD80 as before. In addition, the dye 7-aminoactinomycin D (7-AAD; BD Biosciences, Pharmingen, San Diego, CA, USA) was added to the cells for viability assessment. Next, the cells were resuspended in DPBS^−/−^, 1% FBS, 2 mM EDTA, and 0.005% NaN_3_ and flushed through a 70-μm cell strainer to create single-cell suspensions. MHC-II^high^ CSF1R^low^ and MHC-II^low^ CSF1R^high^ chBMDC subsets were sorted by FACS with a BD Influx cell sorter, equipped with 405-, 488-, 561-, and 635-nm lasers (BD Biosciences, Pharmingen, San Diego, CA, USA). Each chBMDC subset constituted close to 25% of the original sample. Approximately 2 × 10^6^ cells were sorted for both subsets to perform quantitative real-time PCR (RT-qPCR). In parallel, the cells were analyzed before and after FACS using the CytoFLEX LX flow cytometer. Moreover, some cells were sorted onto a Polysine® microscope slide, fixed with 4% paraformaldehyde, and analyzed by confocal microscopy using the TCS-SPE-II microscope to confirm the expression patterns of MHC-II, CD80, and CSF1R by the chBMDC subsets. In addition, chBMDCs were sorted to evaluate the phenotypic stability of the cells by prolonged cell culture. Sorted chBMDCs were seeded into 24-well plates at 350,000 cells per well in 1 ml of RPMI culture medium with GM-CSF and incubation for 1 or 3 days of prolonged cell culture at 41°C, 5% CO_2_, before repeated flow cytometric analysis.

### Gene Expression Analysis of Separated chBMDC Subsets Using RT-qPCR

Sorted chBMDC subsets were lysed in RLT buffer (Qiagen GmbH, Hilden, Deutschland) and stored at−20°C until RNA isolation. RNA isolation was performed with the RNeasy Mini Kit (Qiagen GmbH, Hilden, Deutschland) according to the manufacturer's instructions, including a DNase treatment step using the RNase-Free DNase Set (Qiagen GmbH, Hilden, Deutschland). Next, cDNA was prepared using the reverse transcriptase from the iScript cDNA Synthesis Kit (Bio-Rad Laboratories B.V., Veenendaal, the Netherlands) according to the manufacturer's instructions. RT-qPCRs were performed with primers (listed in Table 2) and either FAM-TAMRA-labeled TaqMan probes combined with TaqMan Universal PCR Master Mix or SYBR Green Master Mix without probes (all from Life Technologies Europe BV, Bleiswijk, the Netherlands). RT-qPCRs were performed with a CFX Connect and analyzed with the CFX Maestro software (both from Bio-Rad Laboratories B.V., Veenendaal, the Netherlands). All RT-qPCRs were evaluated for proper amplification efficiency (95–105%) using serial dilutions of reference cDNA from splenocytes that were stimulated with concanavalin A for 24 h or from HD11 cells that were stimulated with LPS for 3 h. RT-qPCRs were performed *in triplo* for every sample, and the average gene expression levels were expressed as 40-Ct values, as described by Eldaghayes et al. (29). The results were normalized toward gene expression levels of the housekeeping genes *28S* and *GAPDH*.

**Table 2:** All primer and probe sequences are given from the 5′ to 3′ ends.

**Gene**	**NCBI Reference**	**Type**	**Sequences (5^**′**^-3^**′**^)**
MERTK	NM_204988.1	Forward	TGTGGAAGGATGGCAGGGAG
		Reverse	GCACGGATGCTGAATGTAGAGG
ZBTB46	XM_015296613.2	Forward	CTGGACCTGTGGAAGAGGAAAC
		Reverse	CGGTAGTGGGAGGCAATCTC
iNOS	NM_204961.1	Forward	TGGGTGGAAGCCGAAATA
		Reverse	GTACCAGCCGTTGAAAGGAC
TLR4	NM_001030693.1	Forward	GTCCCTGCTGGCAGGAT
		Reverse	TGTCCTGTGCATCTGAAAGCT
GAPDH	NM_204305.1	Forward	GTGGTGCTAAGCGTGTTATC
		Reverse	GCATGGACAGTGGTCATAAG
CD14	NM_001139478.1	Forward	GGACGACTCCACCATTGACAT
		Reverse	GGAGGACCTCAGGAACCAGAA
		Probe	AATGATCTTCCTGATTTGCAGACTGCCAA
CCR6	XM_015284122.2	Forward	GCCAGCCGCAGAAGAATGTA
		Reverse	TGTGGAGAAGAGTTTCAGAATGCT
		Probe	CAGAGTCGTGCAACATCGTCTGACCTACA
CCR7	NM_001198752.1	Forward	CATGGACGGCGGTAAACAG
		Reverse	TCATAGTCGTCGGTGACGTTGT
		Probe	TGAGGGTCACCATCGCTTTCAGCC
DEC205	NM_001037836.1	Forward	AACACGATGCCAGCTCTCAA
		Reverse	TTGACATGAAACGTAAGCTTCCTT
		Probe	CTACCAGTTCAACACCCAGTCTGCTCTTTCTTG
DC-SIGN	NM_205484.1	Forward	TCTCGCTGAGCAGAATGAGTTG
		Reverse	GATGAGGTGGGAGTGCATCTC
		Probe	CACAAAGCGAAGGCGGAGTGCG
CD83	XM_418929.6	Forward	TTGGCGACAGAATAGCATGG
		Reverse	CAGGGAGCCTCCAAGTCCTT
		Probe	AAGTCCTTGATGTGGAATCTCGTCATCCA
28S	XR_003078040.1	Forward	GGCGAAGCCAGAGGAAACT
		Reverse	GACGACCGATTTGCACGTC
		Probe	AGGACCGCTACGGACCTCCACCA

*In the absence of probe sequences, quantitative real-time PCR (RT-qPCR) was performed with SYBR Green Master Mix*.

### Mixed Lymphocyte Reaction Between chBMDC Subsets and PBMCs

PBMCs were isolated from heparinized blood by density gradient separation using Ficoll-Paque PLUS according to standard procedure and resuspended in X-VIVO 15 cell culture medium. The cells were seeded in a 96-well flat-bottom culture plate with 100,000 cells per well. Unsorted and sorted MHC-II^low^ and MHC-II^high^ chBMDC subsets were resuspended in X-VIVO 15 medium and added to the PBMCs at different effector-to-target (E:T) ratios, ranging 1:2–1:8. A positive control was created by addition of 1 μg/ml of anti-CD3, 1 μg/ml of anti-CD28, and 1:50 supernatant from a COS-7 cells transfected with a pCI-neo construct expressing chicken IL-2. A negative control was created by adding an additional X-VIVO 15 medium. The cells were cultured for 3 days at 41°C, 5% CO_2_. Subsequently, 0.4 μCi of ^3^H-thymidine per well was added to the culture for 18 h, and the cells were harvested using a Harvester. ^3^H-Thymidine incorporation by the cells was determined in a 1,450 MicroBeta Plus liquid scintillation counter.

### Statistical Analysis

Statistical analysis was performed using GraphPad Prism 7 software (GraphPad Software, La Jolla, CA, USA). The data were tested for the assumptions of normally distributed data. Flow cytometry expression data showing the geometric mean fluorescent intensity (gMFI) was log-transformed in order to generate normally distributed data. Paired *t*-tests were used to test for statistically significant differences between MHC-II^low^ and MHC-II^high^ chBMDC subsets. A *p*-value of <0.05 was considered statistically significant.

## Results

### chBMDC Cultures Are Heterogeneous and Comprise MHC-II^low^ and MHC-II^high^ Subsets Cells That Differ in Morphology and Phenotype

**Figure 1:**
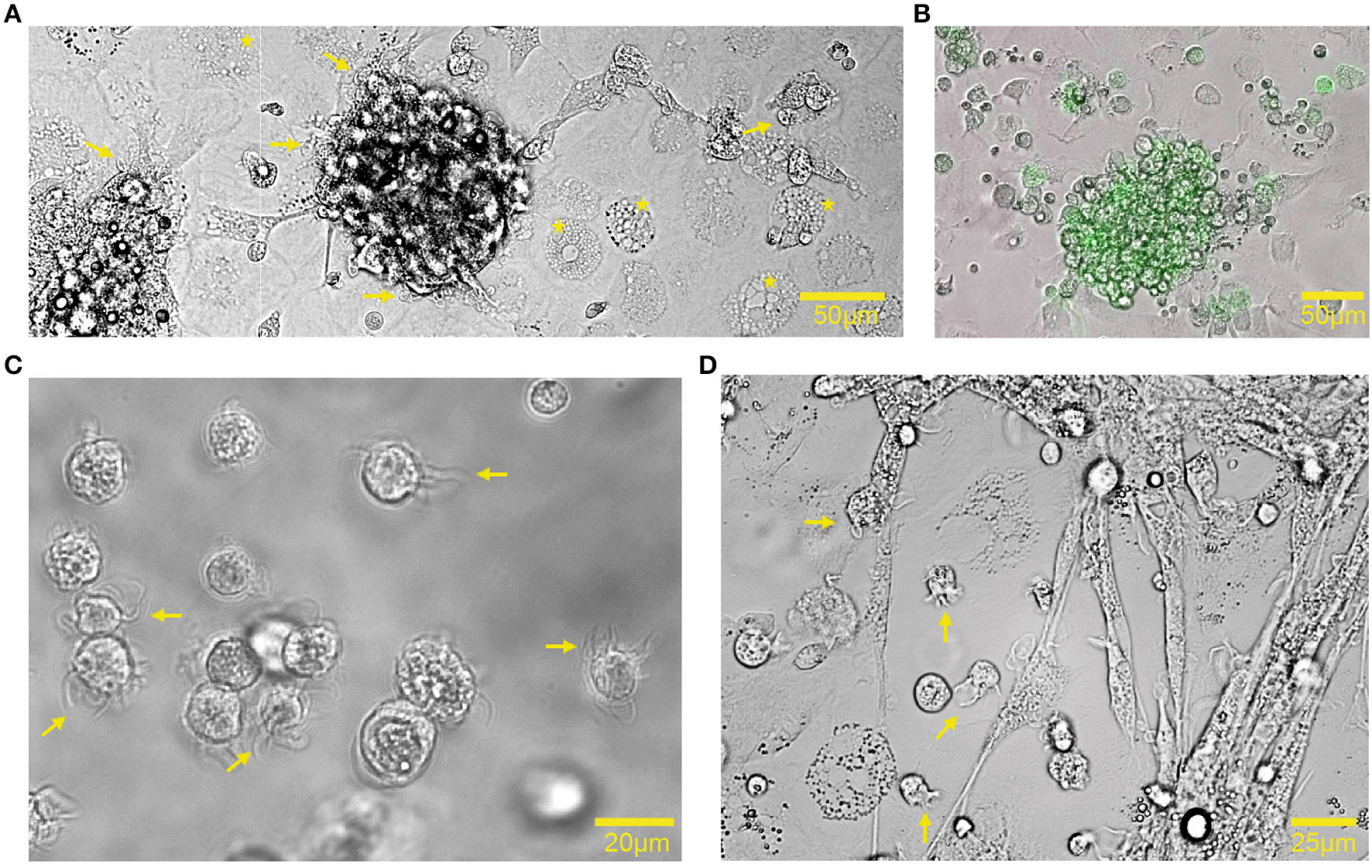
Chicken bone marrow-derived dendritic cell (chBMDC) cultures contained clusters of major histocompatibility complex class II (MHC-II)^+^ cells. **(A)** Bright-field light microscopy shows unstimulated chBMDCs after 7 days of culture. A cluster with chBMDCs is visible at the center. Yellow arrows indicate dendritic cells, visible as veiled cells that show protrusions. Yellow stars indicate highly granular cells without protrusions. **(B)** An overlay of light and fluorescent microscopy shows a cluster of unstimulated chBMDCs with MHC-II expression in green. **(C)** Light microscopy shows individual unstimulated chBMDCs that were transferred to a Petri dish after gentle resuspension in DPBS^−/−^. **(D)** Light microscopy shows lipopolysaccharide (LPS)-stimulated chBMDCs.

Bone marrow cells, derived from bones of 18-day embryonated chicken embryos, were cultured in the presence of GM-CSF for 7 days to generate chBMDCs. The bone marrow isolates comprised mainly myeloid immune cells, including monocytes, thrombocytes, thromboblasts, granulocytes, and erythrocytes (Supplementary Figure 1A). After 7 days of culturing, nearly all cells were found to be CD45^+^ and thus hematopoietic (Supplementary Figure 1B). In agreement with previous studies, chBMDCs formed clusters holding veiled cells (Figures 1A,C, arrows), typical for DC morphology (14), and highly expressing MHC-II (Figure 1B). MHC-II-expressing cell clusters did not appear in the absence of GM-CSF (Supplementary Figure 2). Beyond the clusters, cells with different morphologies were visible. These were large, round, without protrusions, and highly granular (Figure 1A, stars). Upon LPS stimulation, cell clusters became less dense, and many individual small cells with elongated protrusions became visible, a feature typical for mature DCs (Figure 1D, arrows).

**Figure 2:**
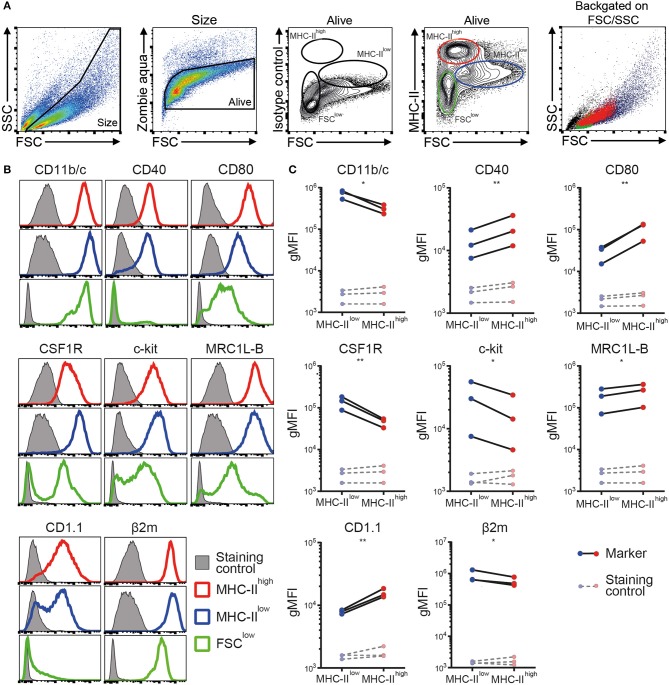
Phenotypic analysis of chicken bone marrow-derived dendritic cells (chBMDCs) shows the presence of different subpopulations. **(A)** The cells were gated, respectively, for their scatter profile [forward scatter (FSC)/side scatter (SSC)], viability (Zombie Aqua), and expression of major histocompatibility complex class II (MHC-II), as shown in the first, second, and fourth panels. The third panel shows the signal after staining the cells with an isotype control antibody that corresponds to the antibody against MHC-II. Three subpopulations were selected based on FSC and MHC-II expression: FSC^low^ MHC-II^−/low^ (green), FSC^int^ MHC-II^high^ (red), and FSC^high^ MHC-II^low^ (blue). The last panel shows the scatter profiles of the different subsets in matched colors, which were overlaid on the first panel. **(B)** Expression levels of a set of phenotypic markers are shown by representative histograms for the three subpopulations in corresponding colors. Each marker addressed was stained with a combination of either unconjugated primary antibody and allophycocyanin (APC)-conjugated secondary antibody or biotinylated primary antibody and APC-conjugated streptavidin. Filled gray histograms represent cells that have been stained with APC-conjugated streptavidin or secondary antibody to show the background fluorescence. **(C)** For the MHC-II^low^ and MHC-II^high^ subpopulations, the expression levels of all phenotypic markers are expressed as the geometric mean fluorescent intensity (gMFI) for three independent replicates. The gray dashed lines represent corresponding controls that have been stained with APC-conjugated streptavidin or secondary antibody alone. Statistically significant differences between chBMDC subsets are shown/indicated by *p < 0.05 and **p < 0.01.

Next, a phenotypic analysis of the chBMDC culture by flow cytometry identified three subsets, distinguished by forward scatter (FSC) vs. MHC-II expression: FSC^low^ with no or low expression of MHC-II (FSC^low^), FSC^int^ with high expression of MHC-II (MHC-II^high^), and FSC^high^ with low expression of MHC-II (MHC-II^low^) (Figure 2A). These chBMDC subsets were evaluated for the expression of myeloid markers including integrin CD11b/c, costimulatory receptors CD40 and CD80, CSF1R, stem cell growth factor receptor c-kit, mannose receptor C-type 1-like B (MRC1L-B), non-classical MHC molecule CD1.1, and MHC class I component β_2_-microglobulin (β2m). FSC^low^ cells showed high expression of CD11b/c, but no expression of CD40 and CD1.1 (Figure 2B). CD80, c-kit, MRC1L-B, and β2m were expressed at moderate levels. CSF1R and c-kit were expressed by some FSC^low^ cells, but not by others, showing further heterogeneity within this subset. Since FSC^low^ cells were largely positive for MRC1L-B but showed no or low expression of MHC molecules and costimulatory molecules, these were likely to represent undifferentiated monocytes. Both MHC-II^high^ and MHC-II^low^ cells showed expression of MHC molecules and costimulatory molecules. Compared to MHC-II^low^ cells, MHC-II^high^ cells expressed higher levels of costimulatory receptors CD40 and CD80, CD1.1, and MRC1L-B (Figures 2B,C and Supplementary Table 1). Conversely, MHC-II^low^ cells expressed higher levels of CD11b/c, CSF1R, c-kit, and β2m. Taken together, MHC-II^low^ and MHC-II^high^ chBMDC subsets both showed a phenotype of antigen-presenting cells, but differentially expressed many myeloid markers.

**Figure 3:**
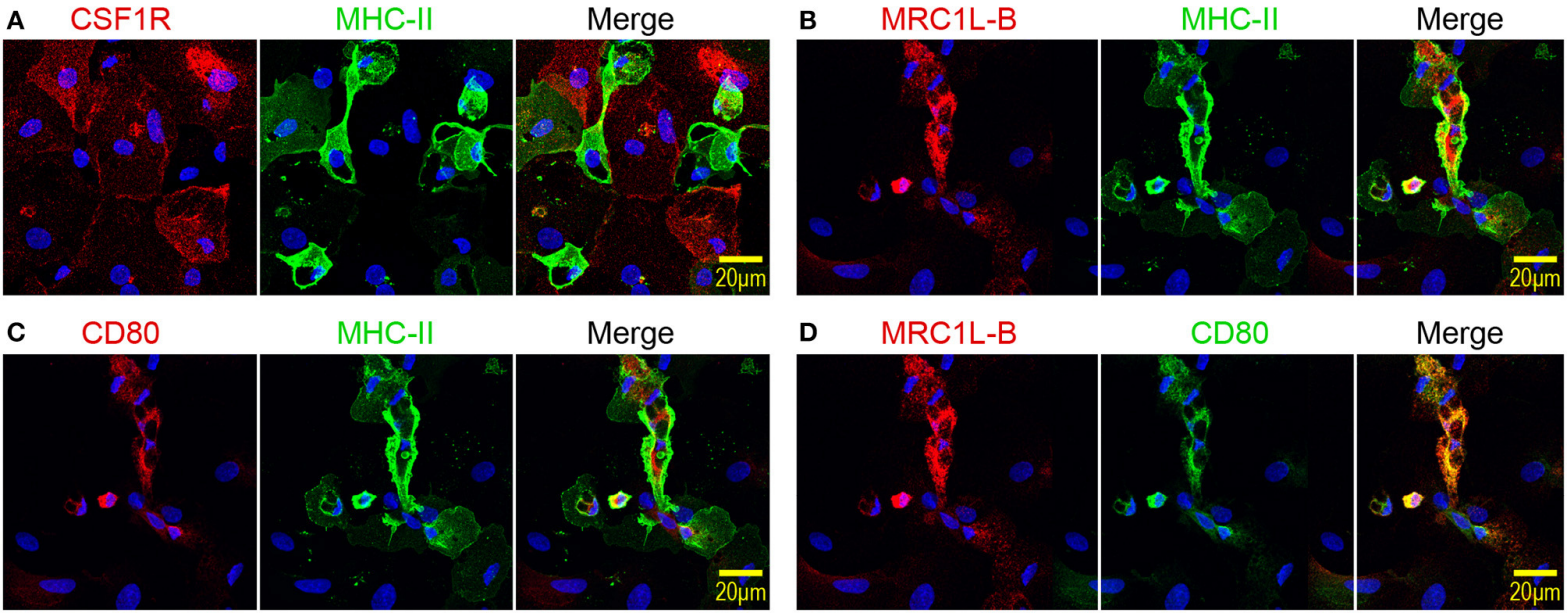
Mannose receptor C-type 1-like B (MRC1L-B) and CD80 are expressed by irregularly shaped MHC-II^high^ chicken bone marrow-derived dendritic cells (chBMDCs), whereas colony-stimulating factor 1 receptor (CSF1R) is expressed by large, round major histocompatibility complex class II (MHC-II)^low^ chBMDCs. **(A)** chBMDCs were stained for expression of CSF1R (red) and MHC-II (green). 4′,6-Diamidino-2-phenylindole (DAPI) (blue) was used as a nuclear staining. A merged image shows potential colocalization between CSF1R and MHC-II. Similar images show the colocalization between MRC1L-B (red) and MHC-II (green) **(B)**, between CD80 (red) and MHC-II (green) **(C)**, and between MRC1L-B (red) and CD80 (green) **(D)**.

Next, the expression patterns of CSF1R, MRC1L-B, and CD80 by MHC-II^low^ and MHC-II^high^ chBMDC subsets were evaluated by immunofluorescent confocal microscopy. The MHC-II^low^ subset expressed higher levels of CSF1R than the MHC-II^high^ subset (Figure 3A), in accordance with the flow cytometry data (Figure 2C). Therefore, CSF1R could be used as an additional marker to discriminate between chBMDC subsets. MHC-II^low^ CSF1R^high^ cells were found to be large and round and to have few protrusions, indicative of a macrophage-like morphology. Similar cells were observed by light microscopy (Figure 1A, stars). In contrast, MHC-II^high^ CSF1R^low^ cells showed irregular shapes with many protrusions, indicative of a DC-like morphology, and resemble the veiled cells that were observed by light microscopy (Figure 1A). MRC1L-B and CD80 expression levels were found to be highest on MHC-II^high^ chBMDCs (Figures 3B,C), in accordance with the flow cytometry data (Figure 2C). Nevertheless, MRC1L-B and CD80 did not colocalize with MHC-II. MRC1L-B and CD80 were mainly found in intracellular compartments, whereas MHC-II was found more on the cellular surface of MHC-II^high^-expressing chBMDCs. A stronger colocalization was observed between MRC1L-B and CD80 (Figure 3D).

### MHC-II^low^ chBMDCs Have a Higher Capacity to Phagocytose Fluorescent Beads Compared to MHC-II^high^ Cells

**Figure 4:**
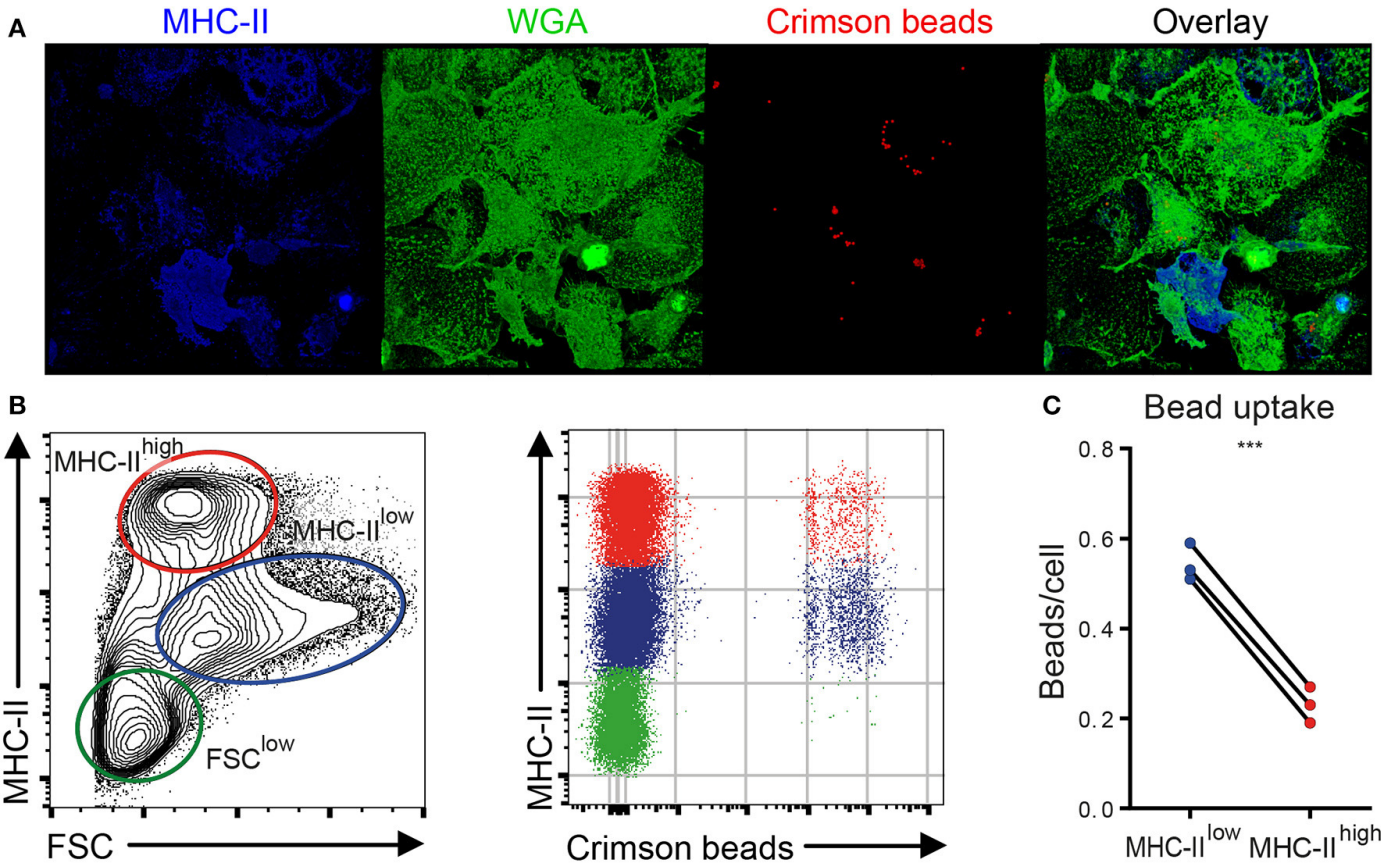
Major histocompatibility complex class II (MHC-II)^low^ chicken bone marrow-derived dendritic cells (chBMDCs) phagocytose IgY-coated beads more efficiently than MHC-II^high^ chBMDCs. **(A)** chBMDCs were stained with wheat germ agglutinin (WGA) to visualize the plasma membrane, shown in green, and MHC-II to identify MHC-II^high^ cells, shown in blue, by confocal microscopy. Fluorescent crimson beads are shown in red. A 3D image was constructed from 56 z-stacks over a total distance of 9.4 μm. Here, a top view on the 3D image is shown, whereas all dimensions are visible in Supplementary Video 1. **(B)** Next, the bead content was quantified by flow cytometry for the previously identified chBMDC subpopulations. **(C)** The average number of beads per cell was calculated for MHC-II^low^ and MHC-II^high^ subpopulations by dividing the mean fluorescent intensity (MFI) of the fluorescent beads by the MFI of cells that contained one bead (visible in **B** as the first positive line of cells). Each line represents an independent replicate (*n* = 3). A statistically significant difference between chBMDC subsets is shown/indicated by ***p < 0.001.

To assess whether the phenotypic distinction between MHC-II^low^ and MHC-II^high^ chBMDCs was functionally relevant, the subsets were assessed for their ability to phagocytose chicken IgY-coated fluorescent latex beads. First, the uptake of IgY-coated crimson fluorescent beads by chBMDCs was confirmed by showing that the beads localize beneath the surface of the plasma membrane, which was visualized using WGA (Figure 4A and Supplementary Video 1). chBMDCs were stained for MHC-II to identify the MHC-II^high^ subpopulation. Both MHC-II^low^ and MHC-II^high^ chBMDCs were found to take up beads as determined by confocal immunofluorescent microscopy. Next, the bead content of the chBMDC subsets was quantified by flow cytometry (Figure 4B). On average, MHC-II^low^ cells (0.54 beads per cell) contained 2.4 times more beads than MHC-II^high^ cells (0.23 beads per cell), which shows that the MHC-II^low^ and MHC-II^high^ chBMDC subsets differ in opsonophagocytic capacity (Figure 4C). A major part of IgY beads actually bound to the cells instead of being taken up, as shown in a separate experiment performed at 4°C (Supplementary Figure 3). However, when bound IgY beads from the experiment performed at 4°C were subtracted from the experiment performed at 41°C, MHC-II^low^ cells were still found to take up 2.7 times more beads than MHC-II^high^ cells. The FSC^low^ chBMDC subset showed little uptake of beads (0.048 beads per cell) (Figure 4B).

### Addition of Recombinant IL-4 to chBMDC Cultures Leads to a Smaller Proportion of MHC-II^high^ Cells

**Figure 5:**
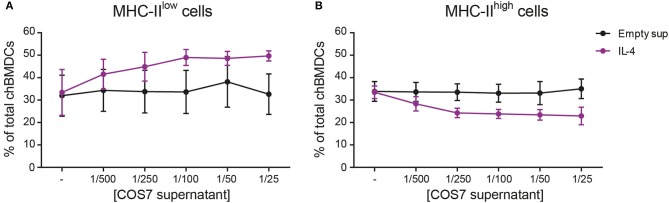
Addition of interleukin-4 (IL-4) to the culture medium during chicken bone marrow-derived dendritic cell (chBMDC) culture reduces the proportion of major histocompatibility complex class II (MHC-II)^high^ cells. The effect of IL-4 on MHC-II^low^ **(A)** and MHC-II^high^ chBMDC **(B)** subsets was assessed by flow cytometry. Supernatant from a COS-7 cell culture transfected with recombinant IL-4 (purple) was used as a source of IL-4, and supernatant from COS-7 cells transfected with an empty vector was used as a control (black). The graphs show the percentages of both chBMDC subsets within total chBMDCs after culturing the cells in the presence of titrated concentrations of IL-4 or empty supernatant control. Both panels show the mean of four independent replicates; the error bars of all panels show the SEM.

The effect of recombinant IL-4 on the generation of chBMDC subsets was investigated, since this cytokine has been used to generate chBMDCs by others (14). To confirm that recombinant IL-4, produced in COS-7 cells, was biologically active, its ability to induce PBMC proliferation was demonstrated (Supplementary Figure 4). Next, IL-4 was given to chBMDCs alone or in combination with GM-CSF. IL-4 alone led to few MHC-II-expressing clusters of chBMDCs (Supplementary Figure 2). IL-4 in combination with GM-CSF led to many MHC-II-expressing clusters, similar to GM-CSF alone. Next, the proportion of MHC-II^low^ and MHC-II^high^ cells was quantified by flow cytometry. The addition of IL-4 to the standard chBMDC culture with GM-CSF was found to increase the proportion of the MHC-II^low^ from 32.6 to 49.7% at the highest administered dose (1/25 dilution) (Figure 5A). The increase in the proportion of MHC-II^low^ cells occurred largely at the expense of the MHC-II^high^ subset, which changed proportionally from 35.0 to 23.0% at the highest administered dose of IL-4 (Figure 5B). The proportion of FSC^low^ cells remained fairly stable (*data not shown*).

### LPS Stimulation Affects the Difference in Phenotype Between MHC-II^low^ and MHC-II^high^ chBMDCs

**Figure 6:**
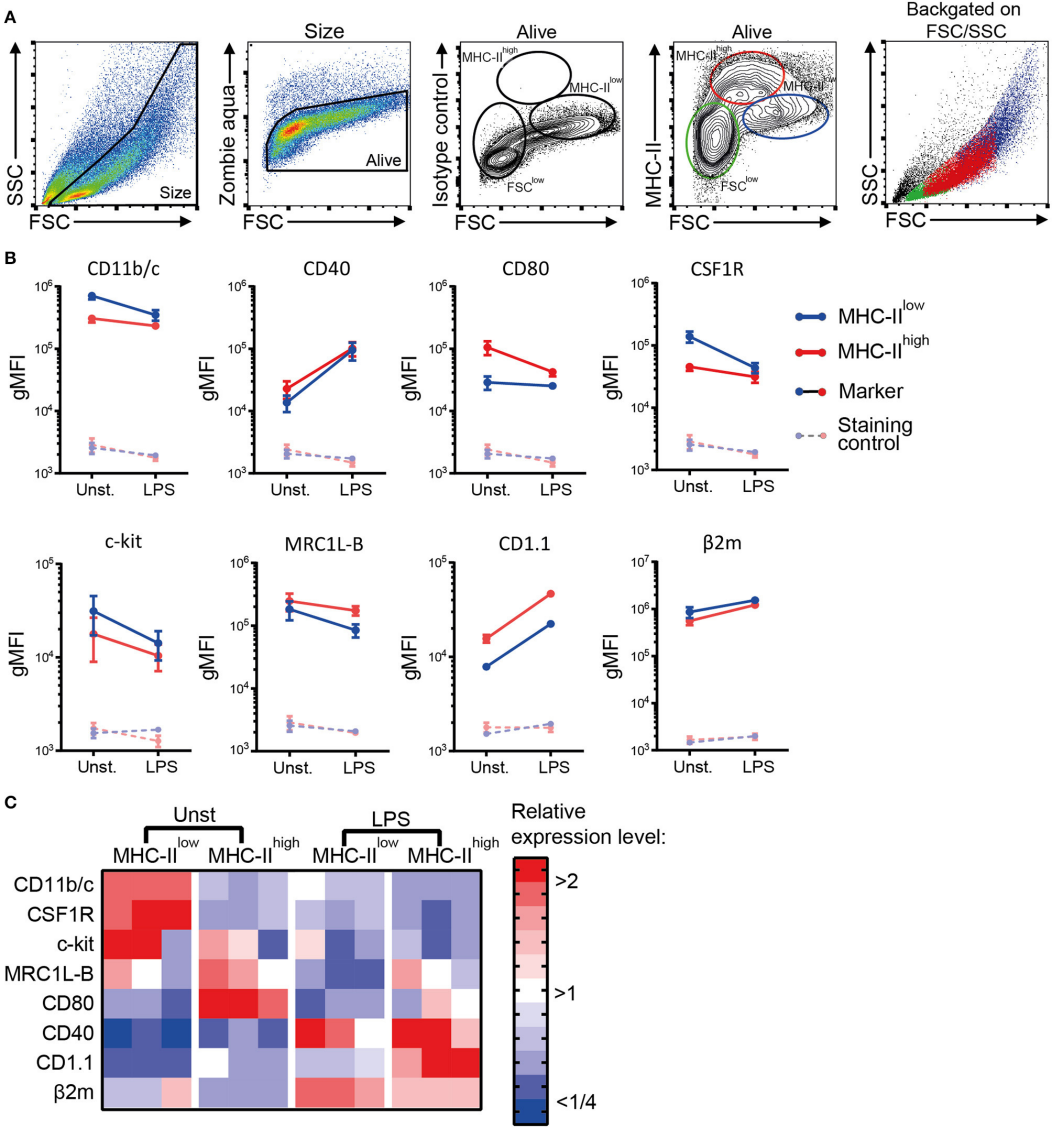
The phenotypic distinction between major histocompatibility complex class II (MHC-II)^low^ and MHC-II^high^ chicken bone marrow-derived dendritic cells (chBMDCs) is not retained after 24 h of lipopolysaccharide (LPS) stimulation. **(A)** LPS-stimulated chBMDCs were gated by a similar strategy as Figure 2A. **(B)** The change in myeloid marker expression upon LPS stimulation is shown for both MHC-II^low^ and MHC-II^high^ chBMDC subsets. The error bars show the SEM of three independent replicates. **(C)** The expression of phenotypic markers is shown in a heat-map for unstimulated and LPS-stimulated MHC-II^low^ and MHC-II^high^ chBMDCs. To obtain the relative expression level for each marker, the gMFI of each sample was normalized to the average gMFI of all samples.

To determine the effect of LPS, commonly used to induce BMDC maturation, the cells cultured for 7 days were stimulated with 100 ng/ml of LPS for 24 h. MHC-II^low^ and MHC-II^high^ chBMDC subsets were still detected (Figure 6A). Both MHC-II^low^ and MHC-II^high^ chBMDCs upregulated CD40, CD1.1, and β2m expression, whereas the subsets downregulated c-kit and MRC1L-B expression (Figures 6B,C). CD11b/c, CSF1R, and c-kit expression on MHC-II^low^ cells decreased to levels similar to those on the MHC-II^high^ subset, while CD80 expression on MHC-II^high^ cells decreased to a level similar to that on the MHC-II^low^ subset. These expression patterns suggest that the phenotypes of the MHC-II^low^ and MHC-II^high^ subsets partially converged. Convergence in expression levels was also observed for CD40 and β2m, but not for MRC1L-B and CD1.1.

### chBMDC Subsets Differ in Maturation Status Rather Than Cell Type

**Figure 7:**
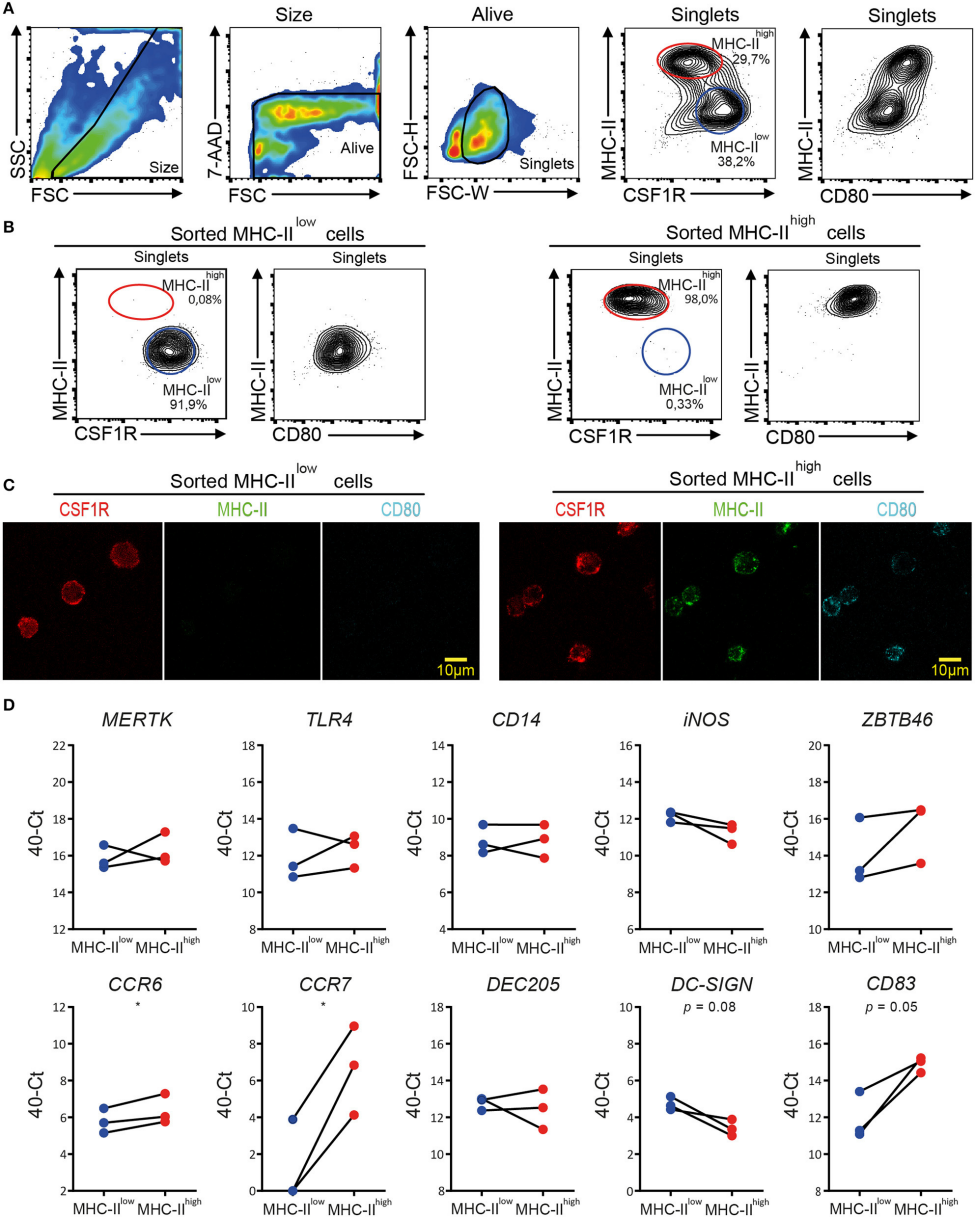
Quantitative real-time PCR (RT-qPCR) on sorted major histocompatibility complex class II (MHC-II)^low^ and MHC-II^high^ chicken bone marrow-derived dendritic cell (chBMDC) subpopulations shows clear differential expression of DC maturation markers, but not of other DC- or macrophage-specific phenotypic markers. **(A)** Pre-sort evaluation of chBMDCs shows the subpopulations that differ in their expression of MHC-II, CSF1R, and CD80. The subpopulations were sorted after gating for scatter profile [forward scatter (FSC)/side scatter (SSC)], viability [7-aminoactinomycin D (7-AAD)], single cells (FSC-W/FSC-H), and expression of MHC-II and colony-stimulating factor 1 receptor (CSF1R). **(B)** Post-sort analysis of sorted MHC-II^low^ and MHC-II^high^ chBMDC subpopulations shows that the sort led to populations of high purity. **(C)** Fluorescent microscopy shows CSF1R, MHC-II, and CD80 expressions for sorted MHC-II^low^ and MHC-II^high^ chBMDCs. Overlays and individual channels are shown with CSF1R in red, MHC-II in green, and CD80 in cyan. **(D)** RT-qPCR was performed on sorted MHC-II^low^ and MHC-II^high^ chBMDCs. The results are expressed at the 40-Ct value with Ct being the number of cycles needed to reach a signal above the threshold. The results were normalized for RNA content by the average expression of housekeeping genes 28 S and GAPDH. Each line represents an independent replicate (*n* = 3). Statistically significant differences between chBMDC subsets are shown/indicated by *p < 0.05. Non-significant *p*-values below 0.10 are given by the actual values to indicate trends close to significance.

MHC-II^low^ and MHC-II^high^ chBMDC subsets were sorted by FACS to determine the differential expression of macrophage- and DC-related genes by RT-qPCR, since well-characterized monoclonal antibodies for these cell surface markers in chickens are scarce. Since MHC-II and CSF1R showed good discrimination between the subsets in confocal microscopy (Figure 3), these markers were used to separate the subsets by FACS (Figure 7A). In addition, chBMDCs were stained for CD80, which was found to be more highly expressed by the MHC-II^high^ CSF1R^low^ subset than the MHC-II^low^ CSF1R^high^ subset (Figure 7A), in accordance with previous results (Figure 2C). Both subsets were sorted to above 90% purity as determined by flow cytometric reanalysis after each sort (Figure 7B). Sorted cells were analyzed by fluorescent microscopy to confirm surface expression patterns of MHC-II, CSF1R, and CD80 (Figure 7C). In accordance with the flow cytometry data, MHC-II and CD80 were expressed by the MHC-II^high^ CSF1R^low^ sorted subset, but not by the MHC-II^high^ CSF1R^low^ subset. In contrast, CSF1R was shown to be present on both sorted subsets. Next, RNA was isolated from the sorted subsets, and RT-qPCR was performed to study the gene expression patterns of macrophage- and DC-enriched genes (Figure 7D and Supplementary Table 2). Tyrosine-protein kinase Mer (*MERTK*), toll-like receptor 4 (*TLR4*), TLR4 coreceptor *CD14*, and inducible nitric oxide synthase (*iNOS*) were used as macrophage-enriched genes, whereas zinc finger and BTB domain-containing protein 46 (*ZBTB46*), C-type lectins *DEC205* and *DC-SIGN*, chemokine receptors C-C chemokine receptor type 6 (*CCR6*) and 7 (*CCR7*), and costimulatory receptor *CD83* were used as DC-enriched genes. Both subsets equally expressed *MERTK* (*d* = 0.46; *p* = 0.60), *TLR4* (*d* = 0.42; *p* = 0.62), *CD14* (*d* = 0; *p* > 0.99), and *DEC205* (*d* = 0.31; *p* = 0.70). MHC-II^low^ cells showed slightly higher expressions of *iNOS* (*d* = 0.90; *p* = 0.16) and *DC-SIGN* (*d* = 1.32; *p* = 0.08), whereas MHC-II^high^ cells showed slightly higher expressions of *ZBTB46* (*d* = 1.48; *p* = 0.24) and *CCR6* (*d* = 0.58; *p* = 0.05). More strikingly, MHC-II^high^ cells showed much higher expressions of *CCR7* (*d* = 5.35; *p* = 0.02) and *CD83* (*d* = 2.98; *p* = 0.05). Thus, the differences between MHC-II^low^ cells and MHC-II^high^ cells were mainly found for DC-enriched genes, especially for *CCR7* and *CD83*, which have been used by others before as maturation markers of chBMDCs (14, 30). In contrast, differences in gene expression were hardly found for macrophage-enriched genes. Combined with the flow cytometric data (Figure 2C) that showed higher expressions of MHC-II, CD40, and CD80 by MHC-II^high^ chBMDCs compared to the MHC-II^low^ chBMDCs, the subsets seem to be DCs at different maturation states rather than different cell types.

**Figure 8:**
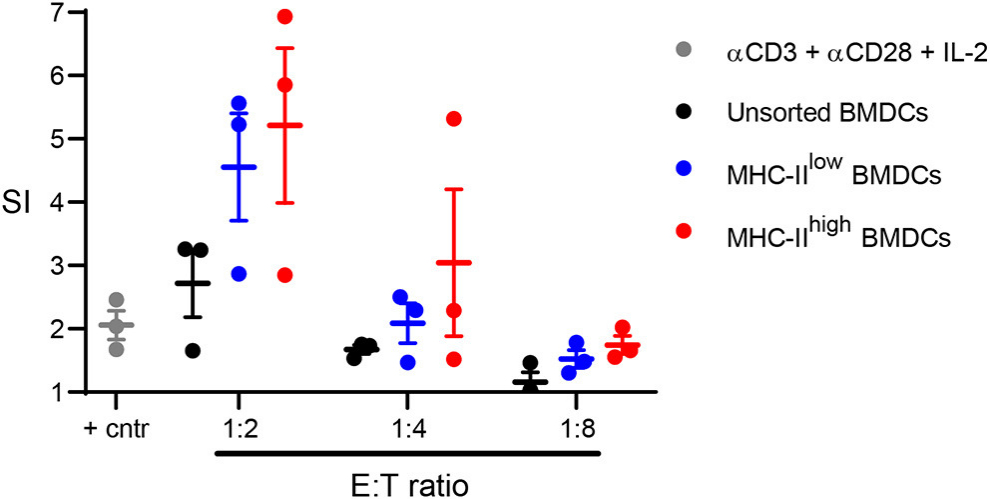
Sorted major histocompatibility complex class II (MHC-II)^low^ and MHC-II^high^ chBMDC subsets induce peripheral blood mononuclear cell (PBMC) proliferation in a mixed lymphocyte reaction. The MHC-II^low^ and MHC-II^high^ chicken bone marrow-derived dendritic cell (chBMDC) subsets were sorted using the gating strategy shown in Figure 7A. Unsorted and sorted chBMDC effector cells were cocultured with PBMCs from laying hens (*n* = 3) at different E:T ratios. In addition, PBMCs were stimulated with a combination of anti-CD3, anti-CD28, and IL-2 as a positive control. After 3 days of culture, proliferation was measured by ^3^H-thymidine incorporation over a period of 18 h and expressed as a stimulation index (SI), which shows the ratio between stimulated and unstimulated PBMCs. All cultures were performed as three replicates.

To determine whether chBMDCs were able to induce T-cell proliferation, allogeneic mixed lymphocyte reactions were performed with sorted chBMDC subsets. Both MHC-II^low^ (SI = 4.6 at 1:2 E:T ratio) and MHC-II^high^ (SI = 5.2 at 1:2 E:T ratio) chBMDC subsets induced proliferation of PBMCs and did so more effectively than unsorted chBMDCs (SI = 2.7) or PBMCs stimulated with a combination of anti-CD3, anti-CD28, and IL-2 (SI = 2.0) (Figure 8).

### MHC-II^high^ chBMDCs Become MHC-II^low^ During Prolonged Incubation After Sorting

**Figure 9:**
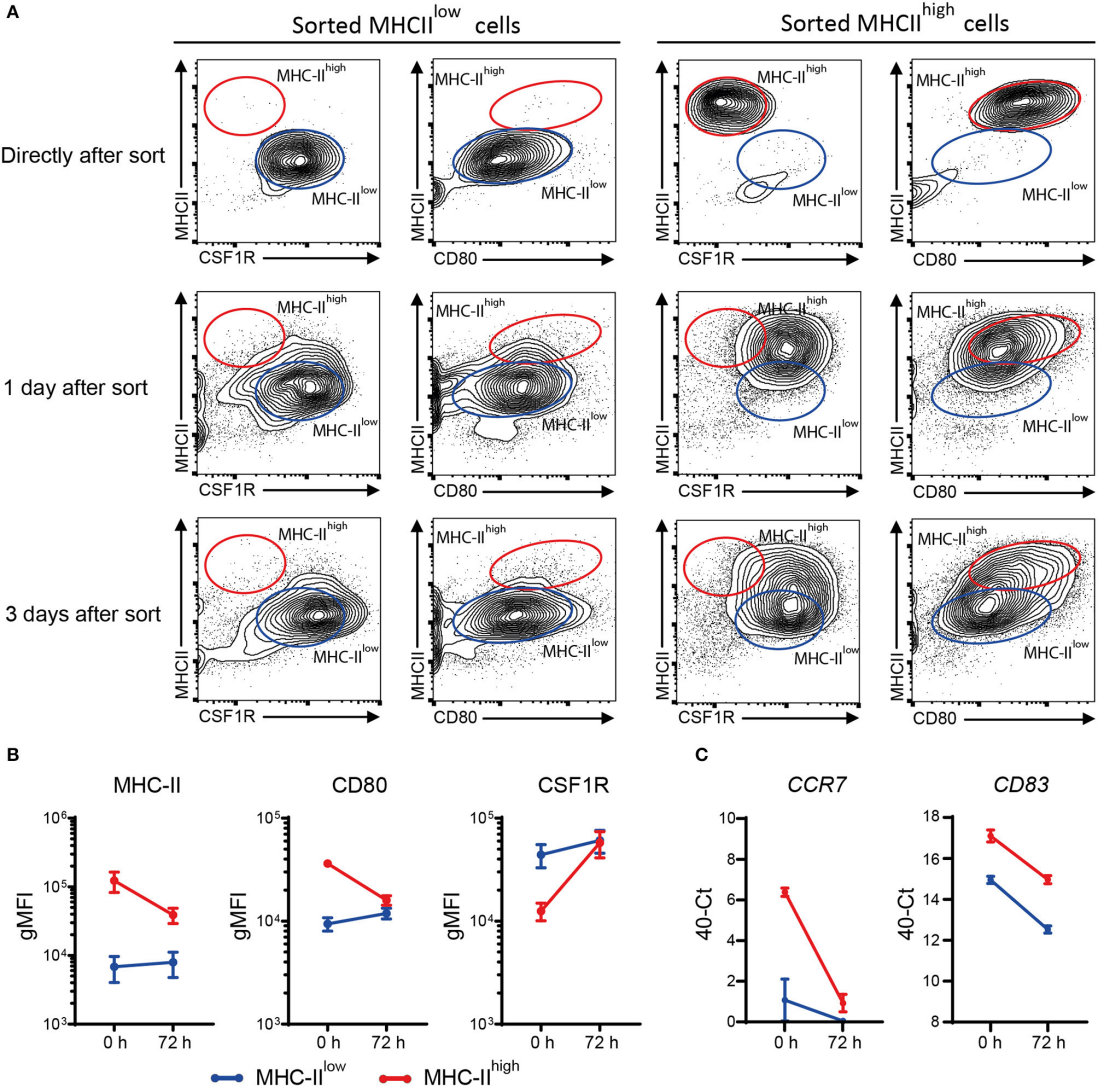
Sorted major histocompatibility complex class II (MHC-II)^low^ chicken bone marrow-derived dendritic cells (chBMDCs) maintain their phenotype, while sorted MHC-II^high^ chBMDCs become MHC-II^low^. **(A)** The MHC-II^low^ and MHC-II^high^ chBMDC subsets were sorted using the gating strategy shown in Figure 7A. The cells were either reanalyzed directly after sorting or seeded for another 1 or 3 days of prolonged cell culture in the presence of granulocyte-macrophage colony-stimulating factor (GM-CSF). After these periods, the cells were stained again using the same protocol that was used before the sort. The contour plots that are shown are representative for three independent technical replicates. **(B)** Surface expression levels of MHC-II, CD80, and colony-stimulating factor 1 receptor (CSF1R) are shown for MHC-II^low^ and MHC-II^high^ chBMDCs directly after sorting (0 h) and after the 3 days of prolonged cell culture (72 h) (*n* = 3). **(C)** Similarly, messenger RNA (mRNA) expression levels of *CD83* and *CCR7* are shown for both subsets at 0 and 72 h (one experiment, performed in triplicate).

MHC-II^low^ and MHC-II^high^ chBMDCs were found to differ in the expression of DC maturation markers, at both the protein and gene expression levels, but were similar in their ability to induce PBMCs to proliferate. To gain additional proof that MHC-II^low^ and MHC-II^high^ chBMDCs represent DCs at different maturation states rather than different cell types, sorted chBMDCs were reseeded for another 1 or 3 days of cell culture and stained again for MHC-II, CSF1R, and CD80. The MHC-II^low^ subset showed only minor changes in the expression of abovementioned markers (Figures 9A,B). In contrast, the MHC-II^high^ subset showed higher CSF1R, lower MHC-II, and lower CD80 expression and consequently became phenotypically more similar to the MHC-II^low^ subset. Therefore, both chBMDC subsets seem to consist of a DC-like cell type, but these appear to be different states of maturation with MHC-II^high^ chBMDCs being at a more mature but reversible state. In addition, the mRNA expression levels of *CCR7* and *CD83* were determined and found to be decreased for both subsets after prolonged cell culture (Figure 9C).

## Discussion

The present study aimed to characterize GM-CSF-induced chBMDC cultures and address their heterogeneity. Despite the widespread use of *in vitro* grown DCs, there is still an ongoing debate about the representativeness of *in vitro* grown DCs for their *in vivo* counterparts (20–22, 24, 25, 31). In research so far, discussion focused mainly on BMDCs from mice and less on those of farm and companion animals. Early studies already described the presence of macrophage and granulocyte “contaminants” in the murine BMDC culture (5, 32). Moreover, murine (20, 33) and ovine (12) BMDC cultures, as well as bovine monocyte-derived DC cultures (34), have been shown to include CD11b^low^ MHC-II^high^ cells with a DC-like phenotype and CD11b^high^ MHC-II^low^ cells with a macrophage-like phenotype. The first study by Wu et al. describing the chBMDC culture already recognized its heterogeneity (14). However, this study excluded adherent and relatively small cells from analysis, which may, respectively, represent the MHC-II^low^ chBMDCs and FSC^low^ undifferentiated monocytes of the current study. In our opinion, these neglected cells should be characterized to interpret responses of the chBMDC culture correctly when analyzed in bulk. This has been illustrated by studies using LPS-stimulated murine BMDCs, in which individual cell subsets (20) or individual cells (35) were shown to respond very differently in their maturation, cytokine expression profile, and capacity to induce T-cell proliferation. Moreover, virus infection studies with chBMDCs, including avian influenza virus (16, 26) and infectious bursal disease virus (36) were analyzed in bulk, while the different chBMDC subsets might vary in their susceptibility for viruses and influence the outcome of these studies.

In the present study, the chBMDC culture was found to contain MHC-II^low^ and MHC-II^high^ subsets, similar to BMDC and monocyte-derived DC cultures in other species (12, 20, 33, 34). Expression of myeloid markers, morphology, and phagocytosis capacity differed between MHC-II^low^ and MHC-II^high^ subsets. Compared to the MHC-II^high^ subset, the MHC-II^low^ subset showed higher expression of CSF1R, contained larger and more granular cells based on flow cytometry scatter profile and light microscopy, and showed a higher capacity to phagocytose IgY-opsonized beads. These results suggested that the MHC-II^low^ chBMDC subset consists of macrophage-like cells, in agreement with studies in murine (20, 33) and ovine (12) BMDCs, as well as bovine monocyte-derived DCs (34). In these studies, macrophage-like MHC-II^low^ cells showed high expression of CD11b, which contributed to the distinction between the MHC-II^low^ and MHC-II^high^ subsets of these BMDC cultures. Unfortunately, this was not possible for the chicken CD11b/c antigen, since the corresponding antibody has not yet been confirmed to recognize either CD11b or CD11c (26). Compared to the MHC-II^low^ chBMDC subset, the MHC-II^high^ subset showed higher expression of costimulatory molecules CD40 and CD80, in agreement with the DC-like phenotype shown for MHC-II^high^ cells in murine BMDC (20, 33) and bovine monocyte-derived DC cultures (34). In addition, the MHC-II^high^ chBMDC subset showed a relatively high expression of the non-classical MHC molecule CD1.1, which is also indicative of a DC-like phenotype (37). Our phenotypical findings of the chBMDC subsets suggested that MHC-II^low^ and MHC-II^high^ subsets, respectively, represent macrophage- and DC-like cells, in agreement with studies that used murine, ovine, and bovine *in vitro* DC cultures (12, 20, 33, 34). However, there is some discrepancy in the literature about murine BMDCs, since its MHC-II^low^ and MHC-II^high^ subsets have also been suggested to, respectively, represent an immature and mature phenotype of the same cell type (3, 38).

To further explore this alternative hypothesis, we investigated whether the chBMDC subsets were different cell types or DCs at different maturation states; MHC-II^low^ and MHC-II^high^ subsets were sorted to perform RT-qPCR for macrophage- and DC-specific markers. Both subsets showed similar expression of the macrophage-specific markers *MERTK, TLR4*, and *CD14*, which is in contrast to studies of murine BMDCs (20, 33, 39). Moreover, no difference in expression of the DC-specific marker *DEC205* was observed, while *ZBTB46* and *CCR6* were only moderately more highly expressed by the MHC-II^high^ subset. The most striking differences between the chBMDC subsets were higher *CD83* and *CCR7* expressions and lower *DC-SIGN* expression for the MHC-II^high^ subset. Increased CD83 and CCR7 expression and decreased DC-SIGN expression have been reported as maturation signatures of human and murine monocyte-derived DCs (20, 40, 41), suggesting that the MHC-II^high^ chBMDC subset represents a mature DC phenotype. Moreover, it has been shown that CCR7 expression is upregulated by chBMDCs shortly after LPS stimulation (36). Taken together, these results suggest that, rather than being different cell types, MHC-II^low^ and MHC-II^high^ chBMDC subsets are DCs at different maturation states with MHC-II^high^ chBMDC being more mature. Nonetheless, MHC-II^low^ and MHC-II^high^ chBMDCs induced similar levels of PBMC proliferation in an allogeneic mixed lymphocyte reaction. Therefore, we hypothesize that of MHC-II^high^ chBMDCs are in a semi-mature state, which is described in literature as the state at which DCs express high levels of MHC-II and costimulatory molecules but do not produce elevated levels of pro-inflammatory cytokines or optimally stimulate T-cell proliferation (42, 43). Reseeding sorted chBMDCs showed that the immature phenotype of the MHC-II^low^ subset remained stable over time, whereas the semi-mature MHC-II^high^ subset decreased MHC-II and CD80 expressions and increased CSF1R expression, indicating the plasticity and reversibility of this semi-mature phenotype. The chBMDC subsets differed in phagocytosis capacity, with the MHC-II^low^ subset being more efficient in bead uptake. This finding can also be explained by a different maturation status of the subsets, since mature DCs generally have a lower phagocytotic capacity (44, 45). Others observed that LPS-induced maturation diminished the phagocytosis capacity of chBMDCs (14), which was not the case for the semi-mature MHC-II^high^ chBMDC subset of the present study.

The semi-mature phenotype of the MHC-II^high^ chBMDCs must have been induced by the culture conditions that were used, since the cells were not intentionally stimulated. Since it is common practice to include IL-4 cytokine in BMDC differentiation protocols of different species, including chBMDCs (10–12, 14, 46, 47), the effect of IL-4 on the development of chBMDC subsets was investigated. When IL-4 alone was added to the culture, we observed that few chBMDC aggregates appeared, in agreement with previous studies (17). Surprisingly, addition of IL-4 led to a lower proportion of MHC-II^high^ cells, inhibiting chBMDC maturation, though the number of observed chBMDC aggregates remained unaffected. To the best of our knowledge, there are no earlier reports that show an inhibitory effect of IL-4 on maturation of the chBMDC culture in terms of MHC-II expression. Previous studies have only observed the occurrence of chBMDC aggregates when investigating the effects of IL-4 (14, 17). Studies with murine and rat BMDCs have reported that IL-4 supplementation leads to proportionally larger MHC-II^high^ subsets (20, 47), in contrast to our findings for the chBMDC culture. Another study has shown that IL-4 has no effect on ovine BMDC yield or phenotype (12). Therefore, the effect of IL-4 on BMDC cultures seems to differ between species. Another parameter affecting BMDCs was the source of the serum used in the culture. FBS was found to lead to a large MHC-II^high^ CD80^+^ chBMDC population (Supplementary Figure 5), whereas chicken serum led to the immature MHC-II^low^ and semi-mature MHC-II^high^ chBMDCs of the present study.

BMDCs are often stimulated by LPS to induce maturation. In the chBMDC culture, LPS stimulation led to a striking increase in CD40 and CD1.1 expression by both chBMDC subsets, whereas expression of maturation marker CD80 by the MHC-II^high^ subset was unexpectedly decreased. Of note, LPS stimulation led to smaller differences between the subsets in their expression of MHC-II, CD80, and CSF1R. Overall, MHC-II^low^ and MHC-II^high^ chBMDCs responded similarly to the LPS stimulus, which favored the hypothesis that the subsets reflected one cell type at different states. In contrast, murine MHC-II^low^ and MHC-II^high^ BMDC subsets were shown to maintain differential gene expression profiles after LPS stimulation, which provided additional proof that the murine subsets were truly different cell types (20).

In conclusion, this study describes the heterogeneity of the GM-CSF-differentiated chBMDC culture, which comprised MHC-II^low^ and MHC-II^high^ subsets that both possess features of antigen-presenting cells. These populations were found to differ in phenotype, morphology, and their phagocytosis capacity, whereas their ability to induce PBMC proliferation was similar. Based on higher expressions of maturation markers MHC-II, CD40, CD80, CD83, and CCR7 by MHC-II^high^ chBMDCs compared to MHC-II^low^ chBMDCs, the MHC-II^low^ and MHC-II^high^ subsets were found to, respectively, represent immature and semi-mature chBMDCs. The semi-mature phenotype of the MHC-II^high^ subset was found to be reversible, since reseeding and prolonged culture of these cells led to a transition toward the immature phenotype of the MHC-II^low^ cells. Taken together, these results yield a thorough characterization of the chBMDC culture and explain its heterogeneity by the simultaneous presence of immature and mature subsets. Our findings are instrumental for the interpretation of experiments that use this culture in future research.

## Data Availability Statement

All datasets generated for this study are included in the article/Supplementary Material.

## Ethics Statement

The animal study was reviewed and approved by the Dutch Central Authority for Scientific Procedures on Animals.

## Author Contributions

RB and CJ designed the research. RB and GA performed the research. RB analyzed the data. VR, WE, and CJ supervised the work. RB, VR, WE, and CJ wrote the paper.

### Conflict of Interest

The authors declare that the research was conducted in the absence of any commercial or financial relationships that could be construed as a potential conflict of interest.

## Supplementary Material

Supplementary Video 1 This shows a 3D constructed model created by confocal microscopy from chBMDCs that phagocytosed fluorescent beads (in red) for 4 h. The cells from the chBMDC culture were stained for MHC-II (in blue) and the plasma membrane by using WGA (in green).
