# Supplementary material for: In vitro Chicken Bone Marrow-Derived Dendritic Cells Comprise Subsets at Different States of Maturation
Source: Front Immunol. 2020 Feb 26;11:141. doi: 10.3389/fimmu.2020.00141 (PMC7054383; doi:10.3389/fimmu.2020.00141)
Supplement: Supplementary file 2 [file Data_Sheet_1.pdf]

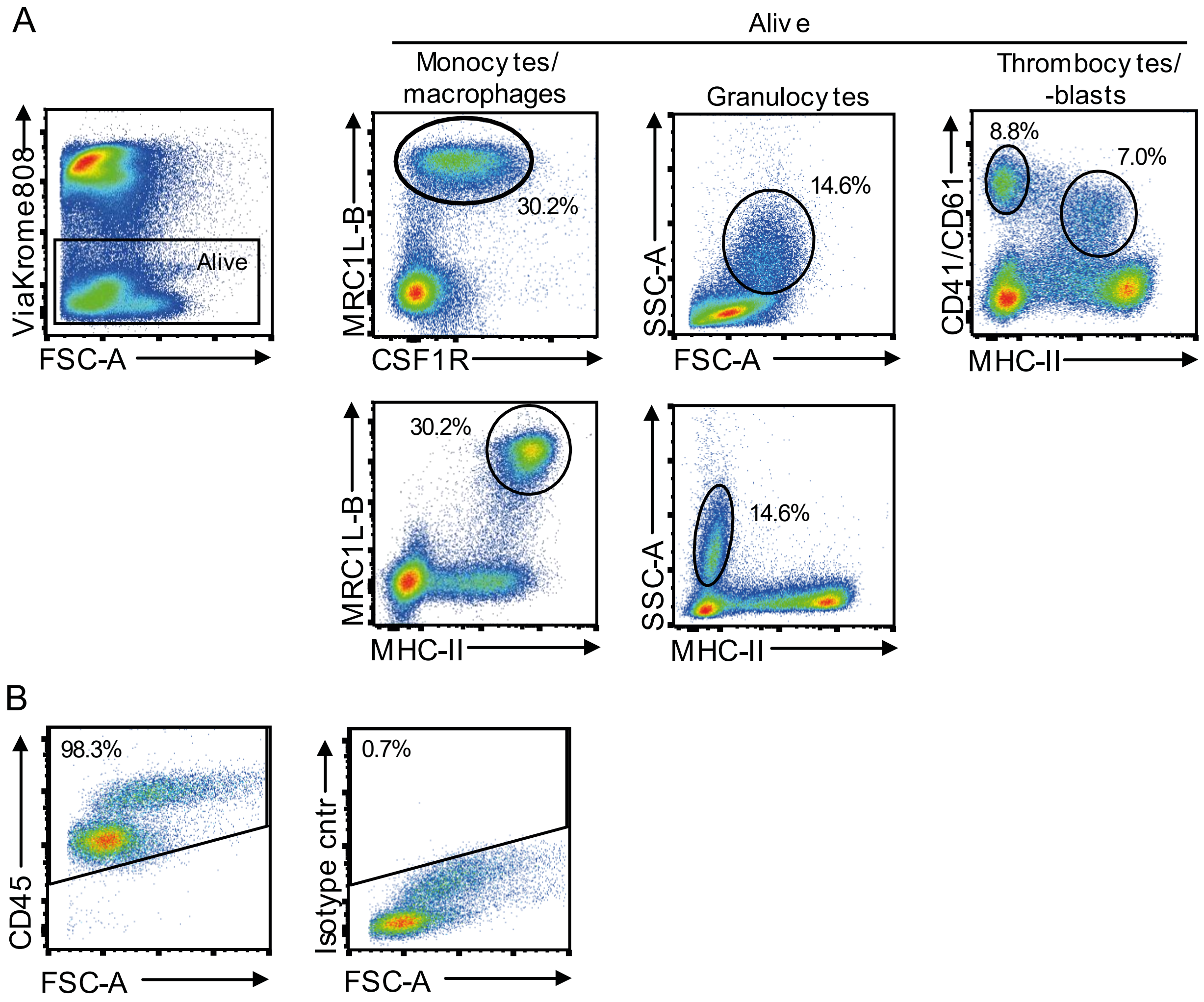

Supplementary Figure 1 Characterization of cells in an embryonic bone marrow isolate. (A) Embryonic bone marrow was characterized by flowcytometry to determine the presence of different subsets of myeloid cells. Cells from the monocyte/macrophage lineage were defined as MRC1L-B^{+} CSF1R^{+} MHC-II^{+}, granulocytes as SSC^{high}, thrombocytes as CD41/CD61^{+} MHC-II^{-}, and thromboblasts as CD41/CD61^{+} MHC-II^{+}. (B) After chBMDCs were cultured for 7 days in the presence of GM-CSF the proportion of hematopoietic cells was determined by CD45 staining.

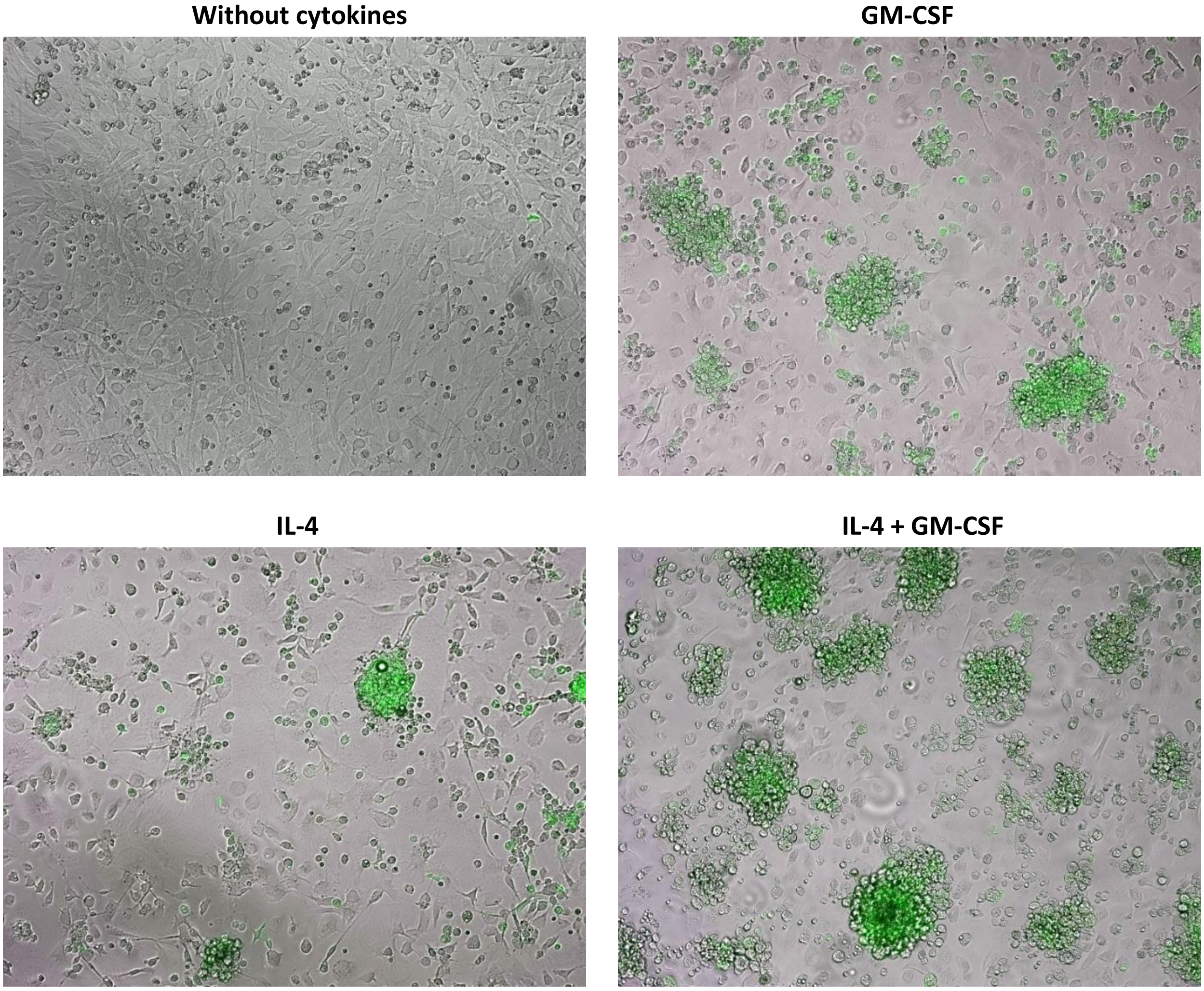

Supplementary Figure 2 The effects of GM-CSF and IL-4 on the morphology and MHC-II expression of the chBMDC culture. The chBMDC culture was performed in the absence of cytokines and in the presence of GM-CSF alone, IL-4 alone, or the combination of GM-CSF and IL-4. COS-7 cell culture supernatant containing the cytokines was added at 2 µl/ml for GM-CSF and at 5 µl/ml for IL-4. The chBMDC culture conditions were one or both cytokines were absent received an equivalent volume of supernatant from a COS-7 cell culture transfected with an empty pCI-neo vector. At the end of the culture (day 8), an AlexaFluor488-conjugated mouse anti-chicken MHC-II monoclonal antibody was added to the cultures at 2 µg/ml and incubated for 30 min at 4°C. Next, the cells were washed twice with DPBS with calcium and magnesium and analyzed using an EVOS FL microscope.

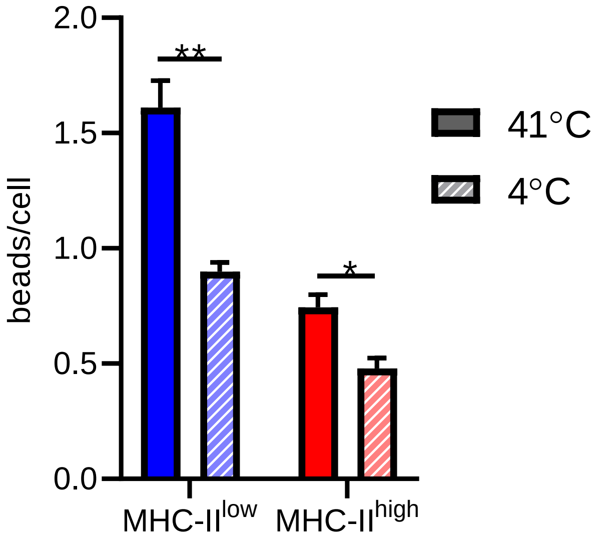

Supplementary Figure 3 Increased phagocytosis of IgY-opsonized beads by chBMDCs upon incubation at 41°C compared to 4°C. IgY-opsonized beads were added to chBMDCs, followed by an incubation period of 4 h at either 41 °C or 4 °C. Next, bead content of MHC-II^{low} and MHC-II^{high} chBMDC subsets was quantified by flow cytometry. The error bars show the SEM of an experiment performed *in triplo*. Unpaired t-tests were performed to test for statistically significant differences between the experiments performed at 41 °C or 4 °C for both chBMDC subsets. A statistically significant difference between the experiments is shown by * for $p < 0.05$ and ** for $p < 0.01$.

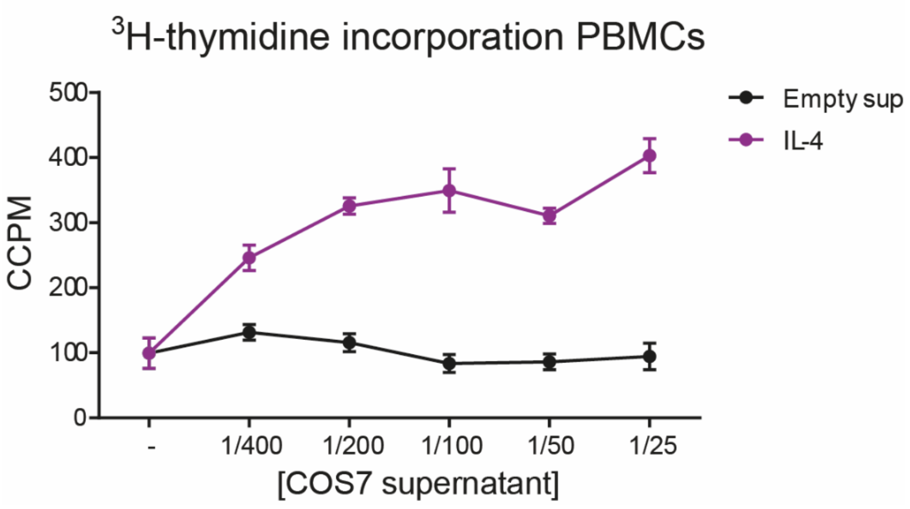

Supplementary Figure 4 IL-4 induced PBMC proliferation. PBMC proliferation was assessed by ^{3}H-thymidine incorporation over a period of 18 h after 4 days of stimulation with COS-7 supernatant containing recombinant chicken IL-4 (purple) or empty COS-7 supernatant as a control (black) (n=4).

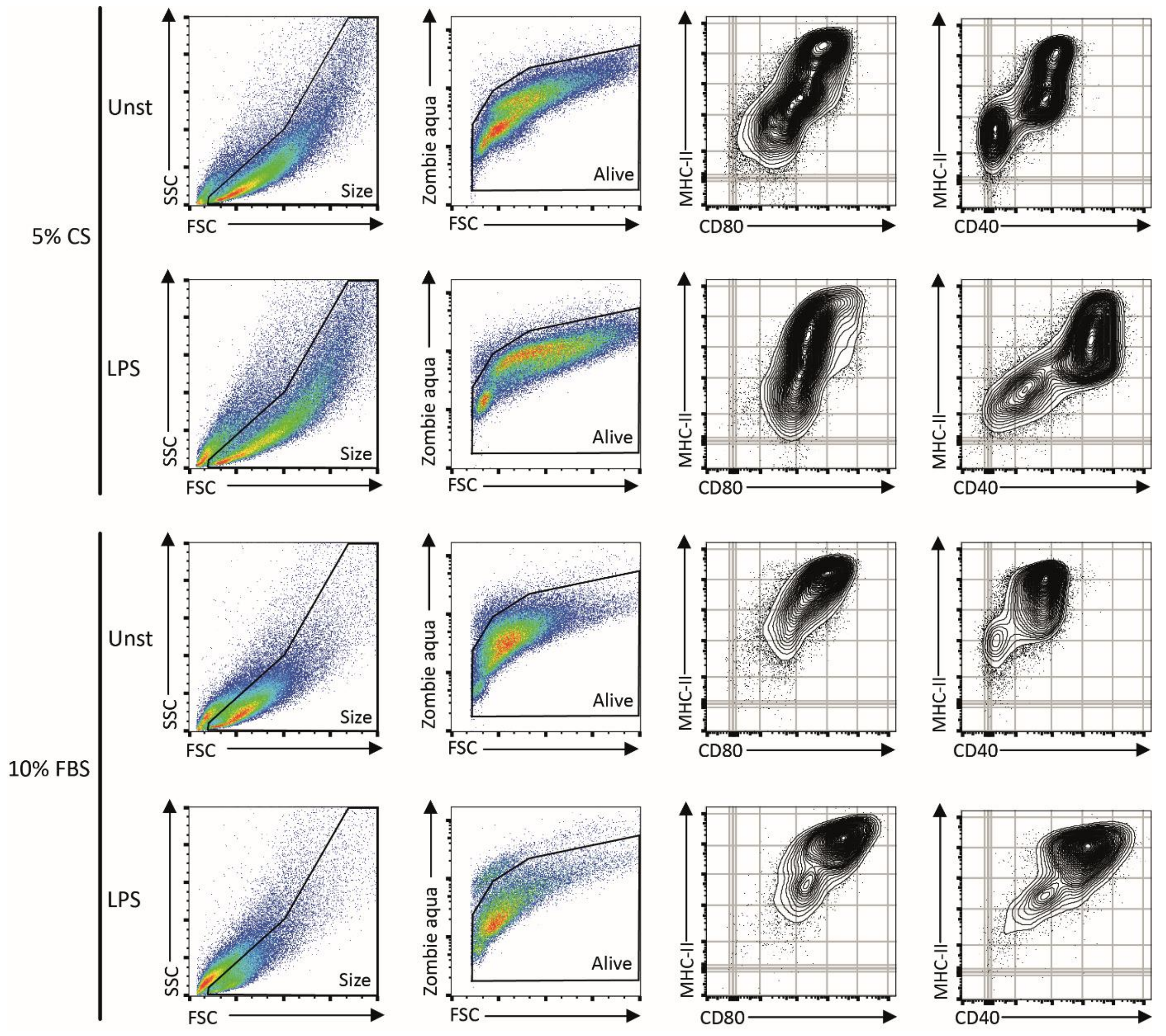

Supplementary Figure 5 The phenotypical outcome of chBMDCs is affected by serum supplements in the cell culture medium. 10% FBS supplemented cell culture medium led to a more homogeneous chBMDC population with higher expression of MHC-II and CD80 compared to chBMDCs grown in cell culture medium supplemented with 5% chicken serum. The FSC^{low} and MHC-II^{low} subsets from the chBMDC culture were almost absent in the adapted protocol that includes FBS supplementation. Moreover, LPS stimulation of chBMDCs in the presence of 10% FBS led to an increase in both CD40 and CD80 expression, whereas only CD40 expression was found to be increased after stimulation of chBMDCs that were cultured according to the standard protocol that includes 5% chicken serum.

Supplementary Table 1 The quantification of flow cytometry data. The average geometric mean fluorescent intensity (gMFI) of three independent replicates is shown for each marker for the MHC-II^{low} and MHC-II^{high} chBMDC subsets. In addition, the ratio between the gMFIs of both subsets are shown for each marker.

Marker	gMFI values per subset		Ratio $MHCII^{low}/MHCII^{high}$
	$MHCII^{low}$	$MHCII^{high}$	
CD11b/c	7.1 x 10^5	3.1 x 10^5	2.31
CD40	1.4 x 10^4	2.3 x 10^4	0.60
CD80	2.9 x 10^4	1.1 x 10^5	0.27
CSF1R	1.4 x 10^5	4.5 x 10^4	3.05
c-Kit	3.1 x 10^4	1.8 x 10^4	1.76
MRC1L-B	1.8 x 10^5	2.4 x 10^5	0.74
CD1.1	7.8 x 10^3	1.6 x 10^4	0.50
β2m	8.6 x 10^5	5.6 x 10^5	1.55

Supplementary Table 2 The quantification of RT-qPCR data. The average 40-Ct-value of three independent replicates is shown for each marker for the MHC-II^{low} and MHC-II^{high} chBMDC subsets. In addition, the difference between the gMFIs of both subsets are shown for each marker.

Marker	40-Ct values per subset		Δ $MHCII^{low}$ - $MHCII^{high}$
	$MHCII^{low}$	$MHCII^{high}$	
MerTK	15.85	16.30	-0.46
CD14	8.823	8.82	0.00
TLR4	11.92	12.34	-0.42
iNOS	12.16	11.26	0.90
Zbtb46	14.02	15.50	-1.48
CCR6	5.78	6.36	-0.58
DEC205	12.78	12.47	0.31
DC-SIGN	4.73	3.41	1.32
CCR7	1.30	6.64	-5.35
CD83	11.93	14.90	-2.98
